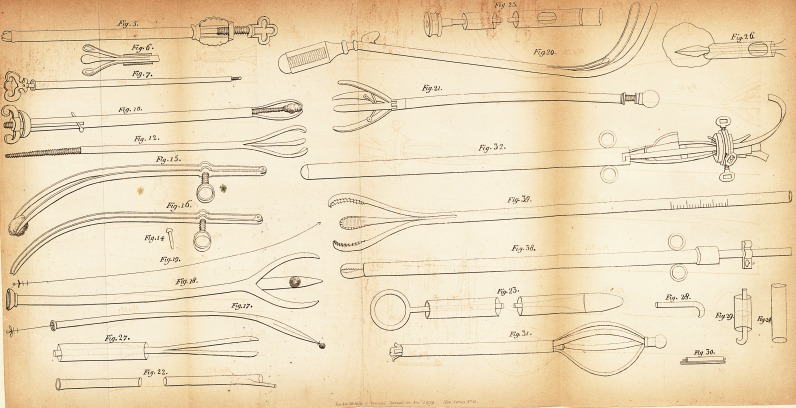# Historical Sketch of Lithotrity

**Published:** 1829-11

**Authors:** W. B. Costello

**Affiliations:** late Assistant to the Inventor, Dr. Civiale.


London Medical & Physical Journal for Novr. 1. 1829. New Series No. 41.

THE LONDON

### Medical and Physical Journal.

N°. 369, VOL. LXII.] NOVEMBER, 1829. [N°. 41, *New Series*.

For many fortunate discoveries in medicine, and for the detection of numerous errors, the world is indebted to the rapid circulation of Monthly Journals; and there never existed any work, to which the Faculty, in Europe and America, were under deeper obligations than to the *Medical and Physical Journal of London*, now forming a long but an invaluable series.—*Rush.*

### ORIGINAL PAPERS, AND CASES,
### OBTAINED FROM PUBLIC INSTITUTIONS AND OTHER AUTHENTIC SOURCES.

### LITHOTRITY.

*Historical Sketch of Lithotrity.* By W. B. Costello, Esq. late Assistant to the Inventor, Dr. Civiale.*

(Concluded from p. 303.)

It was in 1817, while Dr. Civiale was engaged in the study of lithotomy, that he first conceived the possibility of curing calculus without the medium of this cruel operation. This notion acquired plausibility from certain facts, which came to his knowledge during the progress of his researches, and his mind was worked into enthusiasm in pursuit of the means of accomplishing so desirable an object, by the horror with which he had been inspired on witnessing a laborious operation of this kind. But it will be seen how far distant he was at that time from the happy end with which his labours were ultimately crowned, when we learn that, influenced as he was by the reigning problem, "the dissolution of the stone," his first inquiries were directed to the power and applicability of chemical reagents. Well knowing the variety of substances that enter into the composition of vesical calculi, and firmly persuaded that the only rational means of destroying those concretions must be reagents, he had naturally two objects in view, or, in medical language, two indications to fulfil. First, to devise means for procuring small portions of the calculus, by which its exact nature would be determined; secondly, to construct a pouch

* We have given in the plate those instruments only to which M. Costello refers in his paper.—Ed.

or bag, capable of holding the stone, and of resisting at the same time the action of whatever chemical substance might be employed for the destruction of the calculus. He accordingly projected two instruments for these respective purposes.

These instruments were modelled in wood, and though, of course, totally disproportioned to the object in view, and very complicated, they nevertheless gave an idea of the effect they were likely to produce when reduced to proper dimensions, and executed in a more perfect manner. But the difficulty of finding an artist capable of executing his models, as well as the greatness of expense necessarily attendant on it, obliged him to apply to the minister of the interior for France, for pecuniary aid.

This application to the minister was made in July 1818, and was accompanied by a memoir, entitled "Details of a Lithontriptic," and contained also drawings of three instruments, of which the description follows:

The first consisted of a cylindrical metallic tube, of a proper thickness, about three lines and a half in diameter, and eleven inches long. On its external surface were four longitudinal grooves, which became perfect channels, by the tube being received into another metallic tube, somewhat larger, but less thick, than the former.* Four branches were fastened by means of hinges to the vesical extremity of the inner tube; each branch being formed of two pieces, in like manner joined together by means of a hinge. Flexible wires of proper sizes descended through each of the channels already mentioned, and, passing through a groove formed for them in the branches, were attached to the extremity of the second piece. By this mechanism, which offers to a certain extent some analogy with that which regulates the movements of flexion and extension in the fingers, the branches were made to open or close at pleasure. The part of the instrument just described was destined to enter the bladder, and may therefore be called its vesical extremity. At the other extremity was fixed the mechanism which enabled the operator to seize, secure, or let go the stone, by acting on each wire singly, or upon all four at once.

The hollow or cavity of the inner or branch tube was destined to receive a perforator, or steel rod, twelve or thirteen lines long, bearing a saw or trepan head. This head

* A modification of Civiale's present instruments was presented to the Royal Academy of Sciences, in the month of February, 1826, by M. Heurteloup. It is established on this principle, and has not produced the effects its author expected from it.

was concealed by the branches when they closed. A handle attached to the other extremity of the perforator facilitated its movement on the stone, while, at the same time, it prevented its head from passing beyond the branches, so that it could not wound the bladder.

This instrument, the mechanism of which is very complicated, was intended for seizing and fixing large stones only. It bears some resemblance to Franco's quadruple. In the preceding part of this article, we viewed the subject as if Civiale had been acquainted with the labours of his predecessors. This view we thought it right to take; for, whether he was aware of them or not, they were facts which enriched science long before him. We can however affirm, from our near connexion with the inventor, that he was totally unacquainted with Franco's quadruple, as well indeed as with every thing else of this nature that had appeared either in distant or more modern times, when he projected this instrument. It cannot, therefore, be regarded in any other light than that of a coincidence.

The principle of the second, or pouch instrument, was the same as that of the first; it was, however, much more simple. In lieu of four, it consisted of two branches only, which being joined at the extremity, resembled a purse clasp. The branches, like those of the instrument already described, were articulated and grooved, so as to lodge the upper edges of the purse. Instead of one central conduit, there were two, the one communicating with the pouch, and destined to convey into it the solvent fluid; the other directly with the bladder. During the operation the urine might escape through this canal; or if, by accident, any portion of the reagent escaped from the pouch, a liquor capable of neutralizing its noxious influence on the coats of the bladder, might be quickly injected through it.

Five screws were necessary to regulate the movements of the first instrument, as each of the branches might be moved independently of the other three; a single one sufficed for the second, as the branches acted simultaneously.*

The third instrument sketched and described in the memoir, differed from the two former in the great simplicity of its mechanism. It consisted of two metallic tubes, of the same length as the former ones, and made to glide one on the other. The substance of the inner tube was much thinner, so that the size of the stilette, or perforator, which

* This project has since been revived by M. Thibaut and M. Robinet, Pharmacien in the rue de Beaume; but without any result.

passed through it was much greater. The external tube was open at both extremities; the internal supported six elastic steel branches, slightly curved at the end.

A strong button screw, fastened at the other end, was made to control the movements of the tubes upon each other.

The stilette, or lithotriteur, was a long steel rod, which passed in the hollow of the branch tube. Its vesical extremity resembled a trocar. The other was received into a handle, by which its action on the calculus was facilitated, at the same time that it limited its introduction into the tube to the length of the branches; a precaution indispensable for the protection of the bladder. When the instrument was closed, by drawing back the external tube, the branches of the inner tube, no longer compressed together, expanded by their own elasticity. To close the instrument again, it was only necessary to force forward the external tube upon the branches; their extremities were thus approximated, and a round head was formed by their reunion, which rendered the introduction of the instrument into the bladder easy. The analogy which this instrument bears to Hunter's pincers, and the bullet forceps of Alphonso Ferri, will be quickly recognised. It is this instrument, since modified, which M. Civiale now uses, and with which he has effected upwards of *one hundred and forty cures*.

The memoir, as it has been already stated, contained details, drawings, and instructions relative to the use of the instruments above described. The minister of the interior caused it to be forwarded to a commission of the Faculty of Medicine; who appointed the Barons Chaussier and Percy to examine it. The commissaries, however, made no report, and the funds demanded were not granted.

M. Civiale was, therefore, reduced to the painful necessity of either abandoning the project altogether, or of pursuing it slowly to its accomplishment, by devoting to it a part of his own slender income.

And here we cannot sufficiently admire his fortitude and perseverance in boldly encountering the innumerable difficulties which arise, on the one hand, from the coldness and doubt with which new discoveries are too frequently received; and, on the other, from the nature of the subject of his labours.

In the beginning of 1819, he caused the first, the most complicated of his instruments, to be executed, and which was destined for seizing large calculi. M. Faizan was the artist employed on this occasion. The instrument, though very imperfect, was notwithstanding made use of on the dead body.

Here we may be permitted to observe, cursorily, that M. Civiale, already acquainted with the several conditions of the urethra, calculated to favor the application of his instrument, vid. its straightness, (or at least its susceptibility of being rendered straight,) dilatability, &c. not only by the writings of Lieutaud and Montaigu, but by repeated experiments upon himself; a species of exercise which he strongly recommends to all who would excel in the use of the catheter. On this occasion he makes the following remark: "When I see a practitioner precipitately introduce a sound, I conclude he has never practised catheterism on himself."

Without this knowledge, he could not have executed, nor even planned, instruments, which, according to the generally received notions, would have been totally inapplicable. It is, therefore, obvious that M. Amussat's researches had only led him to the knowledge of a fact with which M. Civiale had been already familiar.

The result of his experiments with this instrument, though not completely satisfactory, were so favorable, that M. Civiale no longer entertained any doubt of his ultimate success. These experiments suggested some useful modifications.

We have already said that his second instrument was on the same principle as his first: it was, therefore, an easy matter to form the branches with the double canal, as well as to arrange the mechanism by which the pouch was to open, shut, and fold at pleasure. He now began to be elated with the near prospect of success, and turned his thoughts to the formation of the pouch; when, to his utter disappointment, on consulting his friend, M. Thenaud, one of the most distinguished chemists of the age, he learned that he (M. Thenaud) knew of no substance, whether animal or vegetable, of the thinness and flexibility required, capable of resisting the action of those acids or alkalies which, according to the state of chemical science, it would be necessary to employ for the dissolution of calculus in the bladder. He was consequently obliged to abandon this long-cherished speculation, though not before he had fully confirmed the truth of M. Thenard's assertions by direct and careful experiments, repeated under a great variety of circumstances.

Satisfied, however, that he was developing a novel conception, and that it would ultimately lead to important results, M. Civiale could not consent to abandon it; and accordingly he set about the execution of his third instrument, which was destined to seize and crush small calculi. This instrument, which had been drawn and described with six branches, was reduced to four even before he had it executed.* This instrument was employed without much difficulty on the dead subject; a small stone having been previously introduced into the bladder, which was filled with water. But, in one of the experiments, one of the branches broke. This accident was of but trivial moment in itself: a more serious difficulty arose, from the smallness of the hole made in the stone, which was not entirely reduced to powder or small fragments until after a conside-

* The late Dr. Meirieu, a skilful mechanic, proposed certain modifications of M. Civiale's instrument in 1826, after having assisted at his operations. He first suppressed the crotchets at the extremities of the branches; he added to the perforator two moveable branches, which are extended or closed at pleasure, so that the calculus might be attacked upon a larger surface. This instrument was tried at the Hôtel Dieu of Paris, for in that establishment every new modification had its chance, with a view to discredit Civiale's instrument. This trial was followed by very serious consequences. The stone could not be fixed in the instrument, and the bladder was pinched. A portion of the mucous membrane was torn out by the instrument. A hemorrhage, which lasted three days, was the consequence; there was very high fever, and swelling of one of the testes. This patient was subsequently cut, and died. Other trials of this instrument, which were made in private practice, proved that this instrument had not the necessary solidity.

The Doctor Baron Heurteloup proposed also, in 1826, new modifications of Civiale's instrument. M. Civiale had shown and explained to him the mechanism of his instrument. M. Heurteloup thought that M. Civiale's four-branched pincers might be applicable to the generality of cases. M. Heurteloup's pincers are so constructed that each of the branches may act separately. He has added a very small three-branch pincers, which he calls the *pince servante*, and which he introduces into the principal pincers. The head of the perforator is suppressed, in lieu of which he has substituted a perforator, the direction of which he changes at pleasure.

The instrument of M. Heurteloup, instead of possessing the advantages which he attributes to it, has the following defects: Its size must frequently render its application difficult; the *pince servante* cannot seize large stones, (as is proved by the case of M. Courtois, on whom he made divers fruitless and painful attempts, for upwards of seven months,) nor small fragments. M. Heurteloup was forced to abandon the use of his *improved instrument* at the Hôtel Dieu, and to have recourse to Civiale's instrument for the extraction of small fragments.

At all events this *pince servante* is of no use when the stone lies close to the neck of the bladder; a circumstance by no means unfrequent. The *maitresse pince*, or mistress pincers, (which has changed its name, since its importation into England, for that of *pince à forceps*,) is then opened behind the stone, and consequently the stone cannot be seized by the *pince servante*.

The mobility of the perforator deprives it, to a certain extent, of its necessary solidity. If the stone attacked be flat, the perforator is liable to strike against one of the branches of the pincers, and may thus be broken or twisted, so as to render its extraction impossible, without lacerating the neck of the bladder and the entire length of the urethra.

Finally, this extremely complicated apparatus is not only useless, but inapplicable for the grinding or crushing of small calculi, or the fragments of large ones.

rable number of trials. A new series of experiments was undertaken; and this defect, which at first view seemed fatal, was, after no inconsiderable pains, effectually remedied, and in January, 1820, this instrument was executed with the following modifications: The diameter of the external tube, hitherto of three lines only, was increased to four. The branches of the inner tube were made longer and more flattened, so that their separation became considerably greater. The thickness of the branch tube, as well as the power of the button screw, were also diminished. The size of the head of the perforator was proportionally augmented, and consequently the destruction which it effected of the stone was much more considerable.

It was during a series of experiments made with this instrument that M. Civiale reduced the number of the branches to three: by this disposition it was permitted to increase their strength, without diminishing their power of seizing and securely fixing the calculus; and the perforator was furnished with three teeth, which, when the instrument was closed, were lodged in the interspaces of the branches.

After this accession to their strength, the branches no longer broke. The head of the perforator, now considerably enlarged, became of threefold utility. The destruction of the stone was greater: from its triangular form, when it was drawn within the branches, it there performed the office of a cone, and extended them to a degree far beyond what had been obtained by their own elasticity alone, and thus stones of the size of a small hen-egg came within the scope of the instrument; while it was no longer necessary to drill small calculi, as they were quickly reduced to small fragments by the united pressure of the branches and the large head of the perforator. The teeth upon the internal surface of the branches were now suppressed, and their extremities were curved at unequal lengths, so as to overlap each other when closed. By this disposition, the bladder was secured against the possibility of being pinched, as the largest of the branches remained alone in contact with its walls when it became necessary to close the instrument.

Hitherto the author's attention had been exclusively bestowed on that part of his enterprise which embraced the principal difficulty, namely, the means of seizing and fixing the stone without injury to the bladder. This object being now fully attained, he naturally turned to the arrangement of other details. Thus, for a simple handle, one with a cog-wheel was substituted, and a support, resembling a watchmaker's lathe, was adapted to the instrument, by which the entire apparatus was held with sufficient steadiness during the operation of drilling. The action of the perforator in its progress through the calculus was promoted and equalised by means of a spiral wire spring, shut into a tube, three inches in length. The column supporting this tube was moveable at will, and the power of the spring itself was moderated, or entirely checked by a screw which was made to descend on the steel pivot, by which this spring pressed on the end of the perforator. The instrument, together with the accessory parts just described, was completed in 1820.

The degree of comparative perfection which he had succeeded in bestowing upon his apparatus, now emboldened him to make trial of it upon living animals. Having already made himself expert at seizing, grinding, turning, and reseizing the stone, without injury to the bladder, in the dead body, and being anxious to ascertain the degree of pain the operation might occasion, he instituted with this view a new series of experiments. The results, however, were far from being satisfactory. Considerable difficulty was experienced in introducing into the bladder very small calculi; whilst the distress and agitation produced by the foreign body in an organ not accustomed to its presence, constantly prevented the just appreciation of the pain which the effort to seize the stone might have occasioned. These experiments were made in the country, whither M. Civiale was obliged to retire for the recovery of his health.

In the beginning of 1822, M. Civiale devised means for augmenting the destructive power of the perforator, by causing its head to deviate from the axis of the long steel rod on which it is supported; thus giving to it a slight degree of eccentricity. This increase of its power was of great use. A certain number of experiments was now made on the dead body, with a view to determine the duration of the operation. Calculi formed of uric acid, or oxalate of lime, of ovoid form, and measuring from twenty to twenty-five lines in circumference, attacked with a perforator of three lines and a half in diameter, not eccentric, required half an hour for their destruction and entire extraction. Calculi of the same kind, measuring from thirty to thirty-five lines in circumference, attacked with the same perforator, but rendered slightly eccentric, required an hour and a half's labour for the comminution and complete extraction. Calculi of the same nature, equal in size to that of a large hen-egg, subjected to the action of a perforator, of four lines in diameter, and deviating from the axis of the rod to the utmost degree a straight canula will admit of, required six sittings of half an hour each to produce the same effect.

When stones of inferior compactness were subjected to experiment, the duration varied from a third to a fourth of the time less. The soft calculi were broken by the mere pressure of the branches, and none were found sufficiently hard to resist the power of the instrument. However, when very hard stones were acted on, the progress of the perforator through the stone was slower, the sound produced by its destruction sharper, and the product almost an impalpable powder.

Feeling still how desirable it would be to increase further the destructive power of the drill, another was proposed, the head of which was split, and might be separated, by mechanism, at the other end, acting on a cone which lay concealed between the two blades, to the extent of seven or eight lines. Having thus accomplished this important and arduous undertaking, he caused similar instruments, of lesser diameters, to be executed, decreasing from four lines down to two. This latter instrument is applicable to children of five years of age, and may be used also for the extraction of small stones from the urethra or bladder.

Having enjoyed the advantage of aiding M. Civiale in his long career of brilliant operations, I imagined two modifications of the perforator, suggested by experience, and of which he unhesitatingly approved. The one consists in prolonging its axis, and placing the body of the perforator at a line and a half from its extremity. This is named the shoulder perforator. The prolonged point penetrates the substance of the calculus, and becomes the centre of the excavation; whilst the shoulder, when turned, describes a circle measuring seven or eight lines in diameter. Two perforations effected by means of this drill will excavate a large-sized stone. When a three-branched instrument is armed with a shoulder perforator, on closing upon it, the prolonged point is covered by one of the branches, whilst the body is lodged between the other two; a tooth is sometimes placed on the convexity of the body, opposite to the prolonged point: in this case, the shortest of the branches is grooved or slit longitudinally, to receive it. The advantages of this drill, like that of the *eccentric* drill, (of which it is a modification,) is that it effects twice the destruction of calculus which the simple drill would produce, without augmenting the bulk of the instrument. It is not, however, so valuable as the simple drill for crushing. The other, which I have not yet employed, is destined to enable the operator to glide over or displace a fungus situated at the neck of the bladder. It consists of the ordinary triangular perforator, having one of the angles rounded and polished. When the branches expand, the smooth or toothless side of the perforator is turned towards the fungus, on which it moves without causing it to bleed; and, if the fungus be pediculated, it displaces it, thus arriving at the calculus, which very often lies behind it.

The results obtained from the use of these different instruments upon calculi of a certain size being now fully satisfactory, other instruments with two branches were imagined for crushing or extracting small calculi. One of these was designated by the name of *brise-pierre.** It consisted of an external canula and two demi-cylindrical blades. When the blades were conjoined and passed together into the canula, their flat surfaces were applied to each other, thus forming a perfect cylinder. Their vesical extremities were in a slight degree curved, and, being elastic, they separated on being freed from the compression of the open canula, though not to the same extent as the three-branched forceps. When the blades were joined, the vesical extremity had somewhat of the form of a serpent's head. At the other ends, the convex surfaces of the blades were indented deeply, so as to be moved by means of a cog-wheel. When this wheel was made to act on one of the blades only, the lower blade remained fixed, while the upper one glided on it; and by the same wheel both the blades were drawn towards the open end of the canula, by the insertion of a pin into two holes which were pierced through the blades, so as to correspond with each other. In the first instance, the small stone between the branches, or blades, sustained the action of one blade only; in the second, it was crushed by the mutual pressure of both blades. This instrument is but rarely employed, on account of the difficulty experienced in seizing the calculus with it.† The objection to it is, that it affords the bladder no protection against pinching; an accident which is rendered still more probable by any

* We have already seen that M. Heurteloup reproduced M. Civiale's four-branched instrument. His *brise-pierre* has been also reproduced, under the name of *brise-coque*.

† M. Colombat had a brise-pierre very nearly resembling this executed by M. Weber. The movement, however, is different. M. Rigal has invented another, and M. Amussat's differs from all three. The last of these instruments was tried on M. Carpenter and Dr. Petiet, but without any satisfactory result.

irregularity in the form of this viscus. Moreover, in all cases where it might be applicable the three-branched forceps is equally so, while it is exempt from the objections to which this dangerous and inefficient auxiliary is so subject.

Thus we have followed M. Civiale in his labours, from the beginning to their final and happy accomplishment. We have explained the progressive changes which his instruments have undergone, and given the dates at which those changes were made. We have seen that his first instrument, with articulated branches, was executed in 1820; that subsequently this instrument was laid aside for the pincers with elastic branches, of which he had given a drawing in his memoir to the minister of the interior in 1818; that, in 1820 and 1821, he had made divers successful applications of this instrument both on the dead subject and on living animals. In the two following years, this instrument was still further modified by the addition of means to prevent the water escaping from the bladder through the instrument, as well as to abridge the term of the operation.

His chief labours being now terminated, the next care of Dr. Civiale was to find out a person who would consent to a practical application of his method. Towards the end of 1823, three patients with stone offered themselves. He immediately apprised the Royal Academy of Sciences of his intention to operate; and, on the 13th January, 1824, M. Gentil, the first patient who ever underwent the lithotritic operation, submitted to the use of the instrument at M. Civiale's house, in the presence of the Chevalier Chaussier and Baron Percy,* the commissioners appointed to report the result to the Academy, a great number of surgeons and physicians of eminence, amongst whom were MM. Larrey, Giraudy, Nauche, Luc, Sedillot, &c. After having assisted at this operation, the commissioners, in bestowing on it the name of "*Civiale's operation,*" or "*discovery* of M. Civiale," speak of it in the following terms: "*This discovery is glorious for French surgery, honourable for its author, and consoling for humanity.*"

Since that time a considerable number of calculous patients have obtained relief by this method from the hands

* It will be remembered that these gentlemen had been appointed also to report on his memoir of 1818. They had this memoir in their hands; they had examined his instruments, witnessed the modifications successively made in them, and were at length witnesses of its application. Their report was read and adopted by this learned body on the 22d of March, 1824.

of Dr. Civiale. When I left him in July, the number of patients cured by him amounted to nearly *one hundred and forty*, and he had then *seventeen* patients under treatment.

In this account we have endeavoured to follow, as closely and concisely as possible, the divers changes and improvements which the lithotritic apparatus underwent, during a period of nearly five years. We have purposely omitted many minute details, which would have perhaps only embarrassed the reader. We have seen that the original theory, "the dissolution of the stone," the most impracticable and useless of all those that had been proposed for the cure of calculus, like alchemy, still a dream, a mere problem, led, nevertheless, to the discovery of a process, the most efficient and brilliant within the domain of surgery, a process which, before the lapse of a few years, is destined to supersede all harsh and dangerous methods hitherto in use for removing stone.

This description terminated, we might deem our task complete, as far as the history of lithotrity or the fame of its admirable author is concerned. But, like all important discoveries, it had no sooner been promulgated than divers persons laid partial or exclusive claim to the priority of the invention. It becomes a duty, therefore, to notice the pretensions of all the parties concerned, and to discuss their validity impartially.

In the preceding part of this article, the claims of Dr. Gruithuisen are discussed, and justice (as far as we could render it) has been done to his ingenious labours.

The next claimant is M. Amussat, who imagined that the entire discovery lay in the use of straight sounds.

We have also shown that Dr. Civiale had been familiar with the use of the straight sound long before 1822, the period at which M. Amussat published his notions on this subject.

M. Leroy is the last and only candidate who contests this point of originality with Dr. Civiale.

However, before we enter on the discussion of this gentlemen's claims, it will be proper to define what is meant by the term "inventor." If we mean by it the man who first conceived the possibility of comminuting calculi in the bladder, the invention belongs to Ammon of Alexandria, to Alsaharavius, Sanctorius, Germanus, the monk of Citeaux, Colonel Martin, Gruithuisen, &c.; and in this case the merit is slender indeed. But if we mean the person who has assembled old and forgotten facts,—him who, in studying those facts, has arrived at new and important inferences,—him who, for mere doctrines and theories, has substituted a rational and practical method, then Civiale, and Civiale alone, is the inventor.

M. Leroy, however, claims the invention of lithotrity.*

It was not until 1822 that a note inserted in the medical journals† intimated that M. Leroy thought of comminuting calculi in the bladder, by means of an instrument, of which a drawing and description is given in his work published in 1825, entitled "Exposé des divers Procédés employés jusqu'à ce jour pour guerir de la Pièrre, sans avoir recours à l'Operation de la Taille." As M. Leroy's pretensions to the invention of lithotrity are founded on this instrument alone, I shall enter into details on this subject, which I shall extract from M. Leroy's work. He gives the follow-

* M. Leroy would wish to be considered the inventor, no doubt: the honour is worthy his ambition. In the month of March 1824, he wrote a letter to M. Civiale, in which he proposes community of rights in the invention of lithotrity.

† "M. J. Leroy has presented an instrument, which he calls *lithoprione*, and which he destines (as its name imports) for sawing calculi in the bladder, as well as for extracting them, without having recourse to the cutting operation, which is so cruel and dangerous. This instrument consists in a straight sound, divided within into five compartments: four of them are disposed around, and give passage to an equal number of watchsprings, which meet on the end of a sound, formed like the button of the instrument of Bellocq. These springs expand or close, at will, in the bladder. The central cavity receives a steel rod, armed with a trepan head, which acts as an *emporte-piece* on the calculus, when it has been secured by the watchsprings. After having perforated it, and made it undergo a loss of substance, it is made to turn and present a new surface, on which the trepan head acts in the same manner; and thus it continues to be changed, until all the fragments, which would be too large to pass through the urethra, are extracted through the cavity of the trepan.

"M. Leroy's instrument might furnish means of utilising the discoveries of modern chemistry. Amongst the reagents capable of dissolving calculi, there are some which may be introduced into the bladder without danger; but, being ignorant of the nature of the calculus, it might be increased, instead of being dissolved.|| This lithiprione, by making known the intimate composition of the stone, will enable us with certainty to select the reagent capable of destroying it; but this advantage, which is certainly great, is however only secondary: there are other results, which it would appear reasonable to expect, and which depend on the action of the instrument itself. These are, the possibility of seizing calculi, were they even the size of a hen egg, of reducing them to powder, and of extracting them from the bladder, without causing to the patient any other pain or fatigue than that occasioned by its introduction, as all the movements of the saw are effected in the interior of the sound.

"M. Leroy would be authorised to expect certain success in the employment of the instrument which he has caused to be executed, if it were possible to make any conclusion from the experiments he has made on dead bodies. Experiments on living bodies will, perhaps, make known to him those defects and difficulties with which he has been hitherto unacquainted."

|| On reading this note, it will be asked, was M. Leroy more advanced in 1822 than M. Civiale was in 1818.

ing description of his instrument: A canula, eight inches long, and three lines and a half in diameter, receives into its cavity another canula, much smaller; between both canula an interval exists, of a quarter of a line at most, in which are placed four watchsprings, tolerably strong, and which are attached to a button. A steel ring, furnished with four screws, serves for the fixing of each of the springs separately. Another ring, armed with a comb (*crête*), which is received into a groove of the canula, serves for maintaining all the four springs together, when the stone is seized." (See Pl.)

This instrument was not proposed by M. Leroy until four years had elapsed from the presentation of M. Civiale's memoir. It is almost unnecessary to add, that the author of it himself abandoned it, not only on account of the danger to which its employment would have necessarily exposed him, but also on account of its absolute inutility for any practical purpose. It is true that this instrument might have been introduced into the bladder, and that the calculus might have been secured by the watchsprings; but it was by no means certain that the calculus could be dislodged from their grasp, should it be rendered necessary. M. Leroy was convinced of the imperfection of this instrument, and he accordingly substituted for the watchsprings an elastic branch pincers. This is shown by the following quotation of Baron Percy's letter to M. Leroy: "I have in my possession one of the little watchsprings, for which you have since substituted the forceps of Franco's relation: you let it drop in my room when you came to show me your instruments; with which, most assuredly, you could *not* have performed one of those brilliant operations of which M. Civiale made us witnesses."*

This substitution, however, was not fortunate for M. Leroy. The branches were not sufficiently curved, nor their extremities sufficiently rounded, to give full security to the bladder; and accordingly, in April 1824, M. Leroy, in making his first application of this instrument, on a female, could not seize the stone. He himself informs us that the bladder was pinched, that great difficulty was experienced in withdrawing the instrument, that the patient was subsequently cut, and that she died.

In M. Civiale's first instrument, there was no provision for preventing the water, previously injected into the bladder, from escaping during the operation. His experiments

* P. 149, op. cit.

on living animals convinced him of the necessity of remedying this defect; and accordingly the instrument is modified to answer this end. M. Leroy's instrument is in the same respect defective: to correct this defect, he copies M. Civiale's instrument.

Thus we see that it was not until several years after M. Civiale had presented his first work on the pulverization of calculi in the bladder, that M. Leroy first spoke of the possibility of this operation. The instrument he presents for this purpose is quite inapplicable. For this he subsequently substitutes another, analogous to that proposed by M. Civiale in 1818;* and, finally, he follows M. Civiale step by step in the rectifications which the latter found it necessary to make in his first instruments.

We shall remark in this place, that the memoir of M. Civiale was presented in 1818; that MM. Chaussier and Percy were appointed reporters on the same; that M. Civiale's experiments were made publicly in the dissecting rooms of the Ecole Pratique;† and that many of his *confrères* were acquainted with his researches: amongst whom we may mention the names of Drs. Alies, Buret, Fenet, Lachaise, Londe, &c., some of whom have since been honourably known in science.‡ It is, therefore, difficult to explain how M. Leroy, who was at that time a student in the School of Medicine of Paris, and who devoted himself especially to the study of this branch of surgery, could have remained ignorant of facts so important, that were taking place, as it were, under his own eyes.

It will appear still more extraordinary that M. Leroy should lay claim to the merit of this invention, when we find him admitting that he had read M. Civiale's manuscript at Baron Percy's house, and "*that he there saw expressed the idea of crushing vesical calculi, by laying hold of them*

* In Fabricius Hildanus we find the drawing of an instrument which resembles the pincers which M. Civiale employed in his first experiments, and which M. Leroy has since adopted. Now it is strange that M. Leroy should quote this author, while he omits reproducing the drawing of this instrument, at the same time that he gives that of several other instruments, which have less analogy with those of which he styles himself the inventor. M. Leroy presents only the *speculum cœcum* of Fabricius Hildanus. It will be asked, was M. Leroy apprehensive, if he reproduced the other instrument, that the resemblance between the pincers he adopted and it would have been too remarkable?

† The school immediately in connexion with the Faculty.

‡ M. Civiale's experiments were made with so little secrecy, that MM. Buret and Lachaise had executed an instrument destined to isolate and attack the calculus in the bladder. In the present year, M. Lachaise made trial of a curved lithotrite of his own invention, in the Hôpital Beaujon, on a patient given to him by M. Blandin, but without success.

*with an instrument resembling the bullet forceps of Alphonso Ferri, and by acting on them by means of a stilette or perforator.*" This avowal ought to set at rest all further controversy on this subject.

M. Leroy has published that he had learned from M. Marjolin, as well as others, that they had seen in M. Civiale's hands the pouch instrument, and none other; and that those gentlemen had never heard of a stilette or perforator; and, notwithstanding, M. Leroy himself informs us that he had read in M. Civiale's memoir of 1818 a proposal of an instrument, *with elastic branches for seizing the stone, and a stilette or perforator for attacking it.* How are these contradictory assertions to be reconciled? Besides, it must be remembered, as I have stated in the London Medical Gazette, that M. Civiale had never executed the pouch instrument, and consequently could not have shown it to any of those gentlemen.

M. Leroy endeavours to take advantage of the fact that the commissioners made no report on M. Civiale's memoir in 1818. Such, however, is the course usually adopted by those learned bodies, not to pronounce on any new method until it has been sanctioned by experience. M. Leroy must be well aware of this. The commission appointed in 1822 to report on his instrument, have not deviated from this course; for, up to 1827, they had not promulgated any report on this subject. The commissioners appointed to examine M. Civiale's memoir in 1818 were the same who were named by the Royal Academy of Sciences to draw up the report in 1824. After alluding, in their report, to this circumstance, and making mention of the drawings and descriptions of instruments contained in this memoir, they express themselves in the following terms: "This lithontriptic apparatus, however, was executed in the following year by a Parisian artist, with the modifications and improvements which it presents at this moment; so that we may trace back, to four or five years since, the method under consideration, although it has not been well known; nor had it acquired complete consistency until somewhat more than three years ago."*

And further on their report, in alluding to the pretensions of M. Leroy, who did not appear until four years later, they say, "It is M. Civiale who arrived the first."

Now, these gentlemen, as we have seen, were in possession of the facts appertaining to this subject for a period of

* Report read before the Academy, 22d March, 1824.

six years. Their judgment was founded on a full knowledge of all the circumstances which bore upon it; and their report is as satisfactory as M. Civiale could have wished it.

At that time M. Leroy raised no murmuring against the report. He confined himself to asserting the merit and independence of his own labours; and this much the commissioners themselves allowed. It was not until the importance of M. Civiale's discovery was confirmed by numerous facts, that M. Leroy put in his demurrer.

M. Leroy felt that he stood in the position of an imitator: to repel the suspicion of having copied from M. Civiale, he published his "Memoire Justificatif," in which he boldly insinuates that the commissioners (two most honourable men) had the base complaisance to connive at a substitution of *authentic pièces*. This disgraceful accusation was made against the venerable Chaussier, and the reporter, Baron Percy. The latter disdained to repel the outrage: his only reproof to M. Leroy was the following paternal letter:

"I entertain no feeling of resentment against you, my dear sir. The only injury you have done has been to yourself; and, had you even injured me, I should have already forgotten it. But how is the contestation you have provoked, to terminate; and whither will it lead you? Your adversary, while you spend your time in reclaiming, proceeds prosperously, enjoying his successes, and does not seem to be conscious of the outcry you are endeavouring to raise.

"He has just now forwarded several signed documents, which he affirms to be authentic, and which I believe to be such: you shall be judged on peremptory proofs, and not on words, which are susceptible of interpretation. I regret vividly that you have engaged yourself in such an affair. Read our report, and you will see that the merit of anteriority belongs to Dr. Gruithuisen, and that M. Civiale only made his appearance ten years after him. He may have had the same idea as Dr. Gruithuisen, in the same manner as I believe it very possible that you conceived the project in litigation, without any communication with either the one or the other. I still have one of the little watchsprings, for which you have since substituted the forceps of Franco's relation: you let it drop in my room, when you came to show me your instruments; with which, most assuredly, you could *not* have performed one of those brilliant operations of which M. Civiale made us witnesses.

"How much I regret that I cannot reconcile you to each other! You are both honourable men; well-informed and zealous physicians. My happiness would be to bring you together amicably. But your article, of I know not what journal, and your printed reclamation, deprive me of all means to do it. I shall perhaps be reduced to the necessity of submitting explanations to the Academy on Monday next, which will not be to your advantage. Behold to what one rash step leads! *But the written proofs which I have to furnish against your pretensions* will not alter the esteem I feel for you, nor abate the attachment for you of one of your oldest predecessors.

(Signed) "PERCY."

"*9th April*, 1824."

In this country, the wording of the programme of prizes for 1826 has been resorted to, in order to maintain M. Leroy's pretensions. The phrase is as follows: "As a title of encouragement, 2000 francs are granted to M. J. Leroy, who, the first, in 1822, made known the instruments invented by him, and which he has since endeavoured to improve." The Academy certainly could not have meant the first lithotritic apparatus which had been invented and executed for the crushing and extraction of calculi from the bladder; for we have seen that the same Academy declared, by its commission, two years before, that "M. Civiale had arrived the first," and that "he had his lithotritic instruments constructed in 1819;" and we have also seen that M. Leroy had a knowledge of M. Civiale's labours, having read his memoir at Baron Percy's house. This memoir contained the descriptions and drawings of three instruments: the first consisted of four articulated branches; the second represented a pouch; and the third was formed of elastic branches, and a stilette or perforator, and had some resemblance to the bullet forceps of Alphonso Ferri. These facts are established by the report of the commission, as well as by the avowal of M. Leroy himself.

I have brought to a conclusion this part of my task, and most assuredly it was not without great reluctance I entered upon it at all. Neither science, nor humanity, nor professional dignity, can derive much advantage from the agitation of questions merely personal. For my own part, I had fully resolved to abstain from such discussions. It was not until it had been attempted to dispossess M. Civiale of the merit due to him, that I felt imperatively called on to defend his just rights. I have, I trust, fully shown how unfair those attacks upon his character have been: their virulence will, I hope, excuse any hasty expressions I may have made use of in the warmth of controversy. It has been to me a really unpleasant duty. I have endeavoured to render justice where justice was due. I was aware, in defending the cause of Dr. Civiale, that I was advocating the cause of truth. I could have had no other inducement.

I did not act from any concealed or personal motive. I have been his pupil and assistant, it is true; but I am not therefore *addictus jurare in verba magistri;* and a pledge of this nature would have been silly in the extreme, if M. Civiale's rights had not been proved to be incontrovertible. I cannot help expressing my regret that, in my endeavours to establish those rights, I have been under the necessity of impugning the pretensions of others.

I entertain no feeling of jealousy against any person whose name is connected with lithotrity. On the contrary, I declare myself, as soon as any real improvement shall have been made in the lithotritic apparatus, not only ready, but eager to adopt it. I bear willing testimony to the high talents of M. Leroy; and I fully concur in the opinion expressed by Baron Percy, that he is a zealous and well-informed physician.

The preceding observations establish,

1. That the divers elements of lithotrity have existed in the remotest times; but that we owe the creation of a rational and applicable method to Dr. Civiale.

2. That Dr. Gruithuisen is not the author of this method.

3. That M. Leroy, whose labours may have been independent of M. Civiale's, is not the author of this method.

I have now redeemed the pledges I made to the profession and the public elsewhere, and no light motive shall induce me to obtrude on either with respect to this controversy in future.

### EXPLANATION OF THE PLATE.

Fig. 5, represents Alphonso Ferri's bullet forceps. This drawing, although it differs much from the one in the author's work published at Lyons in 1553, is the most generally known as Ferri's bullet forceps.

— 6. This bullet forceps, with the branches expanded.

— 7. Ambrose Paré's gimlet, for the perforation of calculi in the urethra.

— 10. Fabricius Hildanus's forceps, for the extraction of calculi from the urethra.

— 12. The internal or branch tube of the same.

— 15 and 16. Daniel Episcopus's pincers, for the extraction of calculi placed beyond the curvature of the urethra.

— 17, 18, and 19. Sanctorius's instrument for the extraction of calculi from the urethra and bladder.

— 20. Sir Astley Cooper's curved forceps for the extraction of calculi from the bladder.

— 21. Franco's Quadrupulus vesicæ.

— 22. Tube proposed by Gruithuisen for irrigating the bladder.

The lateral prolongation at the extremity was destined to keep the calculus from contact with the open tube.

Fig. 23. A thick metallic canula, with a conductor bearing a conoid point to render its introduction into the bladder easy. This conductor is terminated by a ring at the opposite extremity.

— 24. A sound, more than three lines in diameter, which Gruithuisen proposed for young subjects.

— 25. A canula, through which a steel rod descends: this rod passes through several circular metallic plates, which prevent it from vacillating. At the outer end a pully is fixed, at the other the crown of a trephine.

— 26. The extremity of a canula, with a lance-pointed stilette. A wire noose, destined to seize the calculus, is passed through the canula.

— 27. The extremity of a canula, with a stone scissors.

— 28. A crook for the extraction of bougies from the urethra.

— 29. A crooked stone breaker. It was destined to crush small calculi against the open end of the canula.

— 30. Two isolated conductors, for the employment of Galvanism.

— 31 and 32. The instrument with watchsprings, proposed by M. Leroy in 1822.

— 38 and 39. The instrument with elastic branches, adopted by M. Leroy.

## Figures and Tables

**Fig. 5. Fig. 6. Fig. 7. Fig. 10. Fig. 12. Fig. 15. Fig. 16. Fig. 14. Fig. 19. Fig. 18. Fig. 17. Fig. 27. Fig. 22. Fig. 25. Fig. 20. Fig. 21. Fig. 32. Fig. 39. Fig. 38. Fig. 23. Fig. 31. Fig. 26. Fig. 28. Fig. 29. Fig. 24. Fig. 30. f1:**